# Meet-URO Score Validation in Real-world Patients with Metastatic Renal Cell Carcinoma Receiving First-line Pembrolizumab Plus axitinib: A Subanalysis of the Prospective ProPAXI Study

**DOI:** 10.15586/jkc.v12i4.403

**Published:** 2025-10-13

**Authors:** Giulia Airò, Annalisa Guida, Alessio Gili, Alessio Signori, Sara Elena Rebuzzi, Marco Maruzzo, Eleonora Lai, Francesco Pierantoni, Davide Bimbatti, Umberto Basso, Alessandra Damassi, Fabio Calabrò, Linda Cerbone, Claudia Caserta, Grazia Sirgiovanni, Debora Serafin, Orazio Caffo, Sarah Scagliarini, Sergio Bracarda, Sebastiano Buti

**Affiliations:** 1 Department of Medicine and Surgery, University of Parma, Parma, Italy;; 2 Medical Oncology Unit, University Hospital of Parma, Parma, Italy;; 3 Dipartimento di Oncologia, Azienda Ospedaliera Santa Maria of Terni, Terni, Italy;; 4 Department of Life Sciences, Health and Health Professions, Link Campus University Rome, Italy;; 5 Department of Health Sciences, University of Genoa, Genoa, Italy;; 6 Medical Oncology Unit 2, Ospedale Molinette, Azienda Ospedaliero-Universitaria Città della Salute e della Scienza di Torino, Turin, Italy;; 7 Oncologia 3, Istituto Oncologico Veneto IOV - IRCCS, Padova, Italy;; 8 Oncologia 1, Istituto Oncologico Veneto IOV - IRCCS, Padova, Italy;; 9 SC Oncologia, Ospedale San Giacomo, Novi Ligure, Italy;; 10 IRCCS National Cancer Institute Regina Elena Rome, Italy;; 11 Department of Oncology, San Camillo Forlanini Hospital, Rome, Italy;; 12 SC Oncologia Medica, Azienda Ospedaliera Santa Maria della Misericordia, Perugia, Italy;; 13 Unit of Medical Oncology 2, Azienda Ospedaliero-Universitaria Pisana, Santa Chiara Hospital, Pisa, Italy;; 14 Department of Medical Oncology, Medical Oncology, Santa Chiara Hospital, Trento, Italy;; 15 Department of Oncology, AORN Cardarelli, Naples, Italy

**Keywords:** Immunotherapy, Prognostic factors, Prognostic score, Renal cell carcinoma, Tyrosine kinase inhibitor

## Abstract

The Meet-URO score provided a more accurate prognostication than the international metastatic RCC database consortium (IMDC) risk group classification for patients with metastatic renal cell carcinoma (mRCC) by incorporating the pretreatment neutrophil-to-lymphocyte ratio (NLR) and the presence of bone metastases in different settings of the disease. To additionally validate the Meet-URO score on overall survival (OS) in a cohort of mRCC patients treated with first-line pembrolizumab plus axitinib, a post hoc analysis of the observational prospective ProPAXI study was conducted. Progression-free survival (PFS) was also considered. Harrell’s C-index was used to compare the discriminative ability on OS and PFS. Overall, the ProPAXI study included 170 patients. Both the five- and the three-risk group Meet-URO score were evaluated to account for the small sample size. The five Meet-URO risk group score showed a mOS of 27.1 months (*p* = 0.064) and 10.3 (*p* = 0.014) months for group 4 and group 5, respectively, while it was not reached for the other groups (*p* < 0.01). Although a worsening of PFS was observed with increasing the risk group, these differences were not statistically significant (*p* =0.19). Similar results were observed fot the three-risk group Meet-URO score. Both five and the three Meet-URO risk groups showed a better C-index for OS (0.69 and 0.66, respectively) compared to IMDC (0.62) and for PFS (0.60 and 0.59, respectively) compared to IMDC (0.56). These findings suggest that the Meet-URO score may provide more accurate prognostic stratification than IMDC alone in mRCC patients treated with first-line pembrolizumab and axitinib.

## Introduction

In recent years, immune checkpoint inhibitors (ICIs) and tyrosine kinase inhibitors (TKIs) associations have become standard as first-line therapy (1–3) for metastatic renal cell carcinoma (mRCC), showing efficacy across the three international metastatic RCC database consortium (IMDC) prognostic groups (4,5).

However, since not all mRCC patients derive durable benefit from these combinations, identifying more accurate prognostic models remains clinically relevant. As the most frequently applied prognostic classification for mRCC, the IMDC score integrates clinical and laboratory parameters. It was first developed in 2009, when vascular endothelial growth factor inhibitors were the mainstay of mRCC treatment, and was subsequently applied in clinical trials involving next-generation TKIs and ICI-based combinations (6). As a result, multiple ICI–TKI combinations demonstrated survival advantages over sunitinib as the first-line treatment for patients with mRCC, each exhibiting distinct toxicity and efficacy profiles (7–9). Although a survival benefit was reported across all IMDC groups, these trials were not designed to assess outcomes within each category. Moreover, a recent Food and Drug Administration pooled analysis and retrospective studies suggests that the use of TKI monotherapy may still represent a valid option as a first-line treatment in favorable-risk patients (10–12). Consequently, the identification of more precise prognostic models or predictive biomarkers reflecting host immune response and tumor biology remains pivotal for refining patient stratification and guiding treatment decisions.

The Meet-URO score is a valuable prognostic tool, validated in a cohort of 571 mRCC patients treated with nivolumab in second-line and beyond setting (13). This score combines the IMDC prognostic classification with two additional factors: the presence of bone metastases prior to treatment and the neutrophil-to-lymphocyte ratio (NLR) in peripheral blood (14). The Meet-URO score has already demonstrated more accuracy than the IMDC for patients with mRCC taking second- and beyond-line nivolumab, first- and further-lines of cabozantinib, first-line combination of nivolumab and ipilimumab, and in a small cohort of patients treated with ICI–TKI combination in second- and third-lines setting (13–18). The current analysis aims to validate the prognostic value of the Meet-URO score in mRCC patients treated with the first-line ICI–TKI combination of pembrolizumab and axitinib within the prospective observational ProPAXI study (19), and to compare its performance with the IMDC.

## Materials and Methods

The Meet-URO score was calculated using baseline data of patients enrolled in the real-world multicenter ProPAXI trial (19). The study involved seven Italian centers and enrolled adults with mRCC, both clear cell and nonclear cell, treated in the first-line with pembrolizumab plus axitinib. This study was approved by the regional ethical committee (Umbria, Italy; protocol number 20684/21/OV). Clinical and laboratory data were collected from medical records using REDCap electronic data capture tools (20,21).

### Prognostic factors

The IMDC prognostic group, the presence of bone metastases, and complete blood count values for the calculation of the NLR were evaluated at baseline to calculate the pretreatment Meet-URO score (web calculator: https://proviso.shinyapps.io/Meet-URO15_score/). We considered both the five- and the three-risk groups Meet-URO score in our analysis (the definitions are available both in Tables S1 and S2). However, given the smaller sample size of the current ICI–TKI cohort and in line with previous studies, the restricted three-risk group version was defined as the primary stratification method.

### Study endpoints

The primary endpoint for this analysis was overall survival (OS) while the secondary endpoint was the progression-free survival (PFS).

The OS was calculated from the first pembrolizumab plus axitinib administration until death, censored at last follow-up for alive patients. The median follow-up was calculated as the median time from the first administration until death or last follow-up for censored patients. The PFS was also described and defined as the time from the start of the treatment to progression or death whichever occurred first. The disease response assessment was clinician-led following the response evaluation criteria in solid tumors (RECIST 1.1) guidelines.

### Statistical analysis

We summarized the main characteristics of the patients using descriptive statistics. The analysis was restricted to patients with complete data on all pretreatment variables included in the Meet-URO score, applying the weights assigned to each prognostic factor during its development. Missing values for other clinical characteristics were not imputed, and the analysis was conducted on a complete-case basis. Patients were categorized into Meet-URO risk groups based on the three clinical and laboratory variables that constitute the score: the IMDC score, the NLR, and the presence of bone metastases.

The Kaplan–Meier method was used to estimate the OS and PFS survival curves for both the original five-risk groups of the Meet-URO score and the simplified three-risk group version. A multivariable Cox regression analysis for OS and PFS was performed to adjust the Meet-URO score for potential confounders, including variables that showed significant differences (*p* < 0.05) in the multivariable analysis of the ProPAXI study. Hazard ratios (HR) were reported with corresponding 95% confidence intervals (CI).

The Harrell’s C-index was calculated for the Meet-URO score and compared with the C-index of the IMDC score to assess their discriminative abilities for OS and PFS. All statistical analyses were performed using STATA (StataCorp, Stata Statistical Software: Release 18, StataCorp LLC, College Station, TX).

## Results

### Patients’ characteristics

We considered 170 patients affected by mRCC with available data for the evaluation of the Meet-URO score. As reported in the ProPAXI study, most patients were male (68%), and the median age was 62 years (range 33–86 years). Most patients had clear-cell RCC histology (83%) and previous nephrectomy (58%). According to the IMDC score, 18.8%, 62.4%, and 18.8% of patients were classified as favorable, intermediate, and poor risk, respectively. Bone metastases were present in 39% of patients. Further patients’ characteristics and details can be found in the ProPAXI study (19).

### Meet-URO score

According to the five-risk group Meet-URO score, 29% of patients belonged to score group 3, 26% to score group 4, 24% to score group 2, 13% to score group 1, and 8% to score group 5. Based on the restricted version of the score, 55% of patients belonged to group 2 (score of 4–8), 37% belonged to group 1 (score of 0–3), and 8% to group 3 (score of 9).

### Correlation between Meet-URO and IMDC score

The joint distribution of the Meet-URO score and the IMDC risk groups is reported in Table 1. Based on the restricted three-risk group Meet-URO score, patients in group 1 were distributed into the IMDC favorable and intermediate-risk group (51% and 49%, respectively), while in group 2, patients were classified into the IMDC intermediate and poor-risk groups (81% and 19%, respectively). Group 3 consisted entirely of patients from the IMDC poor-risk group.

**Table 1:** Classification of patients by three-risk groups Meet-URO and international metastatic RCC database consortium score.

Prognostic group	IMDC risk group (N (%))			Total
	Favorable	Intermediate	Poor	
1	32 (51%)	31 (49%)	0	63
2	0	75 (81%)	18 (19%)	93
3	0	0	14 (100%)	14
Total	32	106	32	170

IMDC = International metastatic RCC database consortium; *N* = Number of patients.

In contrast, according to the IMDC classification, the favorable-risk group was represented entirely by group 1, the intermediate-risk group was divided between groups 1 and 2 (29% and 71%, respectively), and the poor-risk group was made up of patients from groups 2 and 3 (56% and 44%, respectively).

### Survival outcomes by the Meet-URO score

**Figure 1:**
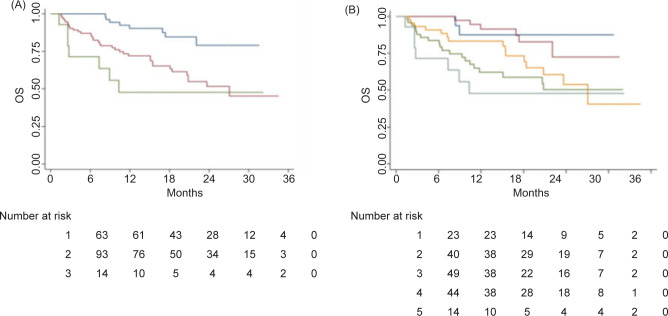
Overall survival according to three- (A) and five-risk groups (B) Meet-URO score.

With a median follow-up of 19.3 months, the three-risk group Meet-URO score demonstrated clear discriminative power (Figure 1A, Table 2). Median OS was not reached for group 1 and was of 27.1 and 10.3 months for group 2 and 3, respectively (*p* < 0.001). The OS estimate for the original five-risk groups Meet-URO score is shown in Figure 1B; in groups 1, 2, and 3, mOS was not reached and in groups 4 and 5, it was of 27.1 and 10.3 months, respectively (*p* < 0.01) (Table 2).

**Table 2:** Cox regression analyses for overall survival in IMDC and five- and three-risk groups Meet-URO Score.

Score	N	mOS (months)	HR	95% CI	*p* Value	C index
IMDC				0.62		
Favorable	32	NR	1.00 (ref)	-	-	
Intermediate	106	NR	3.55	(1.1–11.6)	0.036	
Poor	32	20.8	5.66	(1.6–19.6)	0.006	
Meet-URO score (5 groups)				0.69		
1	23	NR	1.00 (ref)	-	-	
2	40	NR	1.59	(0.3–7.9)	0.568	
3	49	NR	5.52	(1.3–23.6)	0.021	
4	44	27.1	4.03	(0.9–17.6)	0.064	
5	14	10.3	7.22	(1.5–34.9)	0.014	
Meet-URO score (3 groups)				0.66		
1–2	63	NR	1.00 (ref)	-	-	
3–4	93	27.1	3.43	(1.6–7.4)	0.002	
5	14	10.3	5.20	(1.9–14.4)	0.002	

OS = overall survival; IMDC = International metastatic RCC database consortium; N = Number of patients; HR = Hazard ratio; CI = Confidence interval; NR = Not reached.

The C-index for OS for the five- and three-risk groups Meet-URO scores were 0.69 and 0.66, respectively, while the IMDC score had a C-index of 0.62 (Table 2).

**Figure S1:**
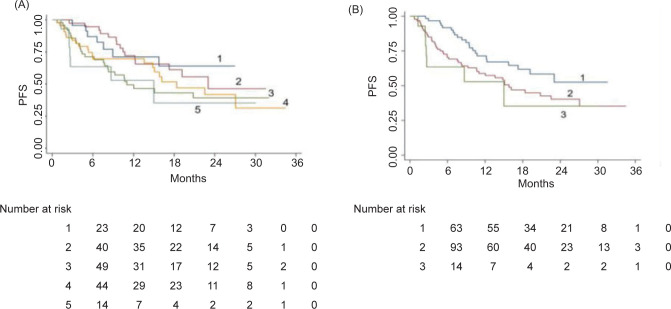
Progression-free survival according to five- (A) and three-risk groups (B) Meet-URO score.

The median PFS calculated for the original five- and the restricted three-risk groups Meet-URO scores are shown in Supplementary Figure S1 and Table S3: although we observed a worsening of PFS with increasing risk group, these differences were not statistically significant (*p* = 0.19 and *p* = 0.06, respectively).

Nevertheless, the prognostic accuracy of the Meet-URO score was also validated for PFS, with a C-index of 0.60 for the five- and 0.59 for the three-risk groups classification, while the IMDC has a C-index of 0.56 (Supplementary Table S3).

### Multivariable analysis for OS

The multivariable Cox regression analysis on OS adjusted for age ≥ 65 years, nonclear cell histology, and gender and adverse events interaction with three-risk group Meet-URO score are reported in Table 3. The three-risk group Meet-URO score retained its statistical significance on OS even after adjustment for the other variables (*p* < 0.01 for group 2 and 3). The multivariable Cox regression analysis including the five-risk group Meet-URO score are reported in Table S4.

**Table 3:** Multivariable Cox regression analyses for overall survival according to three-risk groups Meet-URO score and tumor histology, patients age, and interaction between sex and adverse events.

	Values	HR	95% CI	*p* Value
Histology	Clear cell	1.00 (ref)	-	-
	Nonclear cell	1.75	(0.9–3.3)	0.090
Age	<65 years	1.00 (ref)	-	-
	≥65 years	1.97	(1.1–3.5)	0.023
Interaction female/No AE		2.55	(1.2–5.3)	0.012
Meet-URO score (3 groups)	1	1.00 (ref)	-	-
	2	2.86	(1.3–6.2)	0.009
	3	4.25	(1.5–12)	0.006

HR = Hazard ratio; CI = Confidence interval; AE = Adverse event.

### Multivariable analysis for PFS

At the multivariable Cox regression analysis on PFS, adjusted for nonclear cell histology, ≥ 3 metastatic sites, adverse events, and gender and adverse events interaction, both the five- and the three-risk group Meet-URO score were no longer statistically significant, as reported in Tables S5 and S6.

## Discussion

The Meet-URO score, incorporating NLR and the presence of bone metastases into the IMDC score, has previously demonstrated its prognostic accuracy in patients treated with ICIs and TKIs (13–16). This was also highlighted in a small and heterogenous cohort of mRCC patients receiving second- and third-line ICI–TKI combinations (17).

In the present work, we evaluated the Meet-URO score in the ProPAXI cohort of patients with mRCC receiving first-line pembrolizumab and axitinib, validating its prognostic significance in this treatment context and showing higher accuracy compared with the IMDC score alone. Specifically, the improved discrimination observed in our study reflects the addition of parameters absent from the IMDC, namely, systemic inflammation (captured by NLR) and the presence of bone metastases; these variables reclassify patients within the IMDC favorable, intermediate, and poor risk groups. Elevated NLR has been associated with impaired immune competence and poorer outcomes across several malignancies, while bone metastases are known to identify a biologically aggressive phenotype; the integration of these variables therefore strengthens prognostic accuracy beyond IMDC alone, leading to a more refined stratification. Importantly, Harrell’s C-indices indicated a superior discriminative ability of the Meet-URO score on OS and PFS. However, a direct statistical comparison of predictive performance was not performed, as using a statistic test to compare two Harrell’s C-indices carried a high risk of inflating type I errors (22).

Several limitations should be acknowledged in this study, including the small sample size, the lack of a comparative arm, and the retrospective nature of the study conducted on a prospectively collected cohort, which preclude definitive conclusions about the prognostic value of the Meet-URO score. Also, although the Meet-URO score yielded higher C-index values compared with the IMDC classification, the absolute improvement—particularly in the three-group analysis— was modest. Nonetheless, such trend is consistent with prior publications where the Meet-URO score has systematically shown higher C-index values than IMDC across different therapeutic settings and strategies (Table 4). In our cohort, the three-risk group version of the Meet-URO score was considered the primary analysis, in line with previous studies and given the limited sample size; this approach provided a more robust and clinically applicable stratification. The five-group version was also reported for completeness and comparability with prior publications; however, results for some strata were inconclusive, likely because of the small patient numbers and events per subgroup rather than a true biological difference, and therefore should be interpreted with caution. Another limitation is the potential collinearity between ANC, already included in the IMDC model, and NLR. Nonetheless, while related, these two parameters are not interchangeable: ANC captures neutrophil count, whereas NLR also accounts for lymphocyte count and systemic immune competence. Also, prior studies have shown that NLR retains prognostic significance beyond ANC, supporting its integration into the Meet-URO score (13–18).

**Table 4:** Predictive accuracy of the Meet-URO score (C-index) for overall survival compared to the international metastatic RCC database consortium score in metastatic renal cell carcinoma patients across various therapeutic settings.

Year, Ref.	N	Therapy line	Treatment type	C-index IMDC score	C-index Meet-URO score
Rebuzzi et al. 2021 (13)	571	≥2^nd^	Nivolumab	0.64	0.69
Rebuzzi et al. 2022 (14)	174	2^nd^ –3^rd^	Cabozantinib	0.57	0.64
Rebuzzi et al. 2022 (15)	306	1^st^	Nivolumab + Ipilimumab	0.65	0.73
Damassi et al. 2024 (16)	104	≥1^st^	Cabozantinib	0.62	0.69
He et al. 2024 (17)	72	2^nd^ –3^rd^	ICI-TKI combinations	0.56	0.71
Rescigno et al. 2025 (18)	1418	1^st^	ICI-ICI, ICI-TKI combinations	0.64	0.68
Present study	170	1^st^	Pembrolizumab + Axitinib	0.62	0.69

N = Number of patients; IMDC = International metastatic RCC database consortium.

In spite of these limitations, the Meet-URO score remains a practical and easily applicable tool that, with the integration of additional parameters, allows for a better prognostic stratification of patients.

## Conclusions

This post hoc analysis of the ProPAXI study suggested that the Meet-URO score is a more effective prognostic classification than the IMDC score alone in patients with mRCC receiving first-line pembrolizumab plus axitinib.

The Meet-URO score represents a valuable additional easy-to-use prognostic tool for mRCC patients eligible for this treatment. This analysis lays the groundwork for future research and external validation across different ICI–TKI combinations, as well as comparative studies to further establish the prognostic role of the Meet-URO score in other first-line treatment settings and explore its potential predictive significance (23).

## Figures and Tables

**Table S1:** Prognostic groups of the original Meet-URO score as the combination of biomarker ratio (NLR) and clinical features (IMDC prognostic groups and bone metastases).

Meet-URO score prognostic group (original score subgroups)	Prognostic factors
1 (0–1)	None *or* Bone metastases
2 (2–3)	NLR ≥ 3.2 *or* Intermediate IMDC
3 (4–5)	Intermediate IMDC + (Bone metastases *or*NLR ≥ 3.2)
4 (6–8)	Intermediate IMDC + Bone metastases + NLR ≥ 3.2 *or* Poor IMDC *or* Poor IMDC + (Bone metastases *or*NLR ≥ 3.2)
5 (9)	NLR ≥ 3.2 + Poor IMDC + Bone metastases

NLR = neutrophil-to-lymphocyte ratio, IMDC = International Metastatic RCC Database Consortium.

**Table S2:** The restricted three-risk group version of the Meet-URO score.

Restricted Meet-URO score prognostic group (original score subgroups)	Prognostic factors
1 (0–3)	None *or* Bone metastases *or* NLR ≥ 3.2 *or* Intermediate IMDC
2 (4–8)	Intermediate IMDC + (Bone metastates or NLR ≥ 3.2) *or* Intermediate IMDC + Bone metastates + NLR ≥ 3.2 *or* Poor IMDC *or* Poor IMDC + (Bone metastates or NLR ≥ 3.2)
3 (9)	NLR ≥ 3.2 + Poor IMDC + Bone metastases

NLR = neutrophil-to-lymphocyte ratio, IMDC = International Metastatic RCC Database Consortium.

**Table S3:** Cox Regression Analyses for PFS in five- and three-risk groups Meet-URO Score

Score	N	mPFS (months)	HR	95% CI	*p* Value	C index
IMDC						0.56
Favorable	32	NR	1.00	–	–	
Intermediate	106	17.3	1.71	(0.9–3.4)	0.121	
Poor	32	15	1.98	(0.9–4.4)	0.092	
Meet-URO score (5 groups)						0.60
1	23	NR	1.00	–	–	
2	40	23.0	1.17	(0.5–2.9)	0.729	
3	49	11.0	2.07	(0.9–4.8)	0.088	
4	44	18.4	1.7	(0.7–4)	0.223	
5	14	15.0	2.48	(0.9–7)	0.090	
Meet-URO score (3 groups)						0.59
1–2	63	NR	1.00	–	-	
3–4	93	15.8	1.7	(1–2.8)	0.041	
5	14	15.0	2.23	(1–5.2)	0.065	

PFS = progression-free survival; IMDC = International Metastatic RCC Database Consortium; N= number of patients; HR = Hazard Ratio; CI = Confidence Interval.

**Table S4:** Multivariable Cox regression analyses for OS according to five-risk groups Meet-URO score and tumor histology, patients age and interaction between sex and adverse events.

	Values	HR	95% CI	*p* Value
Histology	Clear cell	1.00 (ref)	–	–
	Non-Clear cell	1.74	(0.9–3.3)	0.094
Age	< 65 years	1.00 (ref)	–	–
	≥ 65 years	1.95	(1.1–3.5)	0.025
Interaction Female/No AE	–	2.46	(1.2–5.2)	0.019
Meet-URO score (5 groups)	1	1.00 (ref)	–	–
	2	1.21	(0.3–6.1)	0.817
	3	3.07	(1.3–7.2)	0.010
	4	2.66	(1.1–6.3)	0.026
	5	4.28	(1.5–12.1)	0.006

HR = Hazard Ratio; CI = Confidence Interval; AE = adverse event.

**Table S5:** Multivariable Cox regression analyses for PFS according to three-risk groups Meet-URO score and tumor histology, number of metastatic sites, adverse events and gender and adverse events interaction.

	Values	HR	95% CI	*p* Value
Histology	Clear cell	1.00 (ref)	–	–
	Non-Clear cell	2.02	(1.2–3.4)	0.009
N of metastases	1	1.00 (ref)	–	–
	2	1.11	(0.59–2.1)	0.743
	3	1.58	(0.8–3.1)	0.181
	≥ 4	2.08	(1.0–4.3)	0.047
Adverse events occurrence		1.84	(1.0–3.5)	0.063
Interaction Female/No AE		2.47	(1.1–5.4)	0.023
Meet-URO score (3 groups)	1	1.00 (ref)	–	–
	2	1.17	(0.7–2.1)	0.586
	3	1.21	(0.5–3.2)	0.706

HR = Hazard Ratio; CI = Confidence Interval; N = number; AE = adverse event.

**Table S6:** Multivariable Cox regression analyses for PFS according to five-risk groups Meet-URO score and tumor histology, number of metastatic sites, adverse events and gender and adverse events interaction.

	Values	HR	95% CI	*p*Value
Histology	Clear cell	1.00 (ref)	–	–
	Non-Clear cell	2.02	(1.2–3.4)	0.010
N of metastases	1	1.00 (ref)	–	–
	2	1.11	(0.6–2.1)	0.759
	3	1.61	(0.8–3.2)	0.170
	≥ 4	2.12	(1.0–4.4)	0.045
Adverse events occurrence		1.82	(1.0–3.5)	0.069
Interaction Female/No AE		2.46	(1.1–5.4)	0.026
Meet-URO score (5 groups)	1	1.00 (ref)	–	–
	2	0.95	(0.4–2.4)	0.904
	3	1.17	(0.5–2.9)	0.729
	4	1.07	(0.4–2.7)	0.881
	5	1.16	(0.4–3.7)	0.026

HR = Hazard Ratio; CI = Confidence Interval; N = number; AE = adverse event.
